# Supplementary figures and images for: Profiling of the Bacterial Microbiota along the Murine Alimentary Tract
Source: Int J Mol Sci. 2022 Feb 4;23(3):1783. doi: 10.3390/ijms23031783 (PMC8836272; doi:10.3390/ijms23031783)

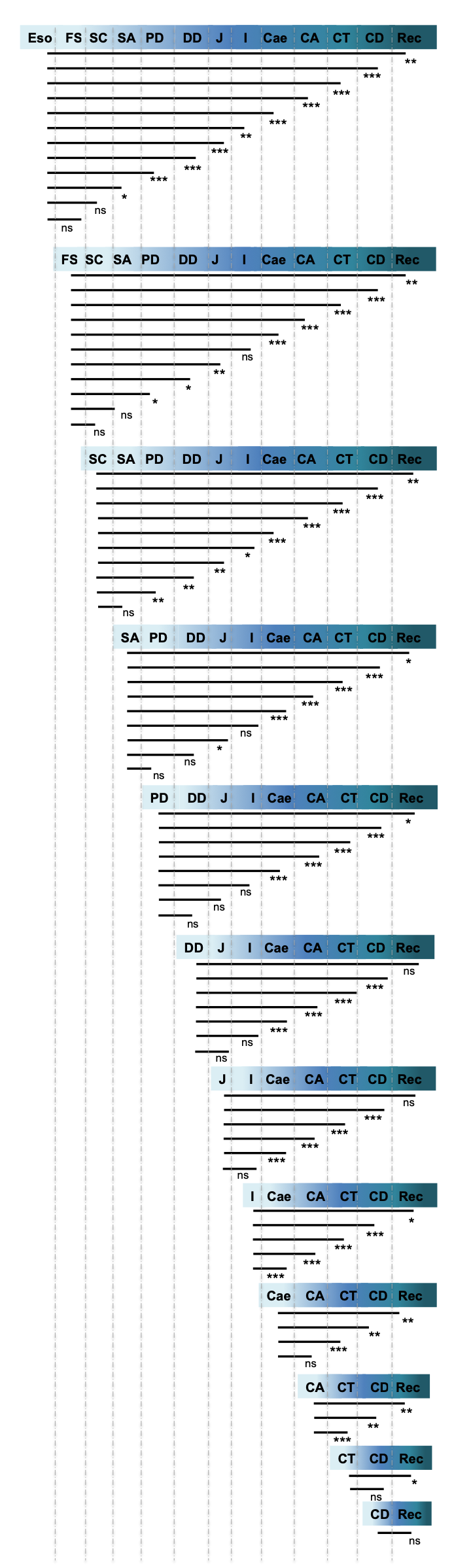

Supplement: Supplementary file 1 [file ijms-23-01783-s001.zip › supplementary file S1.tiff]
